# First human dose-escalation study with remogliflozin etabonate, a selective inhibitor of the sodium-glucose transporter 2 (SGLT2), in healthy subjects and in subjects with type 2 diabetes mellitus

**DOI:** 10.1186/2050-6511-14-26

**Published:** 2013-05-13

**Authors:** Anita Kapur, Robin O’Connor-Semmes, Elizabeth K Hussey, Robert L Dobbins, Wenli Tao, Marcus Hompesch, Glenn A Smith, Joseph W Polli, Charles D James Jr, Imao Mikoshiba, Derek J Nunez

**Affiliations:** 1 GlaxoSmithKline, 5 Moore Drive, Research Triangle Park, NC, 27709, USA; 2 Profil Institute for Clinical Research, Chula Vista, CA, USA; 3 Tandem Labs, Durham, NC, USA; 4 Kissei Pharmaceutical Company LTD, Matsumoto City, Japan

**Keywords:** Remogliflozin etabonate, Sodium-dependent glucose transporter 2 inhibitor, Pharmacokinetics, Pharmacodynamics, Type 2 diabetes mellitus

## Abstract

**Background:**

Remogliflozin etabonate (RE) is the prodrug of remogliflozin, a selective inhibitor of the renal sodium-dependent glucose transporter 2 (SGLT2), which could increase urine glucose excretion (UGE) and lower plasma glucose in humans.

**Methods:**

This double-blind, randomized, placebo-controlled, single-dose, dose-escalation, crossover study is the first human trial designed to evaluate safety, tolerability, pharmacokinetics (PK) and pharmacodynamics of RE. All subjects received single oral doses of either RE or placebo separated by approximately 2 week intervals. In Part A, 10 healthy subjects participated in 5 dosing periods where they received RE (20 mg, 50 mg, 150 mg, 500 mg, or 1000 mg) or placebo (4:1 active to placebo ratio per treatment period). In Part B, 6 subjects with type 2 diabetes mellitus (T2DM) participated in 3 dose periods where they received RE (50 mg and 500 mg) or placebo (2:1 active to placebo per treatment period). The study protocol was registered with the NIH clinical trials data base with identifier NCT01571661.

**Results:**

RE was generally well-tolerated; there were no serious adverse events. In both populations, RE was rapidly absorbed and converted to remogliflozin (time to maximum plasma concentration [C_max_;T_max_] approximately 1 h). Generally, exposure to remogliflozin was proportional to the administered dose. RE was rapidly eliminated (mean T_½_ of ~25 min; mean plasma T_½_ for remogliflozin was 120 min) and was independent of dose. All subjects showed dose-dependent increases in 24-hour UGE, which plateaued at approximately 200 to 250 mmol glucose with RE doses ≥150 mg. In T2DM subjects, increased plasma glucose following OGTT was attenuated by RE in a drug-dependent fashion, but there were no clear trends in plasma insulin. There were no apparent effects of treatment on plasma or urine electrolytes.

**Conclusions:**

The results support progression of RE as a potential treatment for T2DM.

**Trial registration:**

ClinicalTrials.gov NCT01571661

## Background

Type 2 diabetes mellitus (T2DM) is characterized by abnormalities of glucose and lipid homeostasis, which drive secondary micro- and macrovascular complications. Clinical evidence indicates that maintaining glycemic control and reducing postprandial glucose excursions can lower the risk of diabetic complications, e.g. reduce the risk of myocardial infarction, renal disease and retinopathy [1,2]. Despite the availability of multiple classes and combinations of antidiabetic agents, the clinical management of T2DM remains challenging, with the majority of patients failing to achieve and maintain target glycemic levels in practice [3]. There is a continued need for novel therapeutic approaches, particularly those with complementary modes of action that will enable further improvement of glycemic control.

Glucose homeostasis is a complex process controlled by gastrointestinal absorption, tissue utilization, hepatic/renal gluconeogenesis and renal filtration/reabsorption/excretion. Under normal physiological conditions when the glomerular filtrate reaches the proximal tubule, glucose is primarily reabsorbed through the active sodium-dependent glucose transporter 2 (SGLT2) located on the apical or luminal membrane of the epithelial cell in the S1 segment [4-6].

SGLT1 is a high-affinity, low-capacity glucose/galactose co-transporter primarily expressed in the intestine and in the kidney [7,8]. In contrast, SGLT2 is a low-affinity, high-capacity glucose transporter selectively expressed in the kidney. Together, SGLT1 and SGLT2 are responsible for the active reabsorption of glucose across the renal luminal membrane [9,10]. Once reabsorbed by the renal epithelial cell, glucose is transported to the blood by facilitated diffusion via the sodium-independent glucose transporter 2 (GLUT-2). The uptake of glucose in the proximal tubules by SGLT1 and SGLT2 is highly efficient, resulting in complete reabsorption of glucose. In humans, genetic alterations in SGLT2 increase renal glucose excretion (up to 200 g/day) with no apparent adverse effects on renal function or carbohydrate metabolism [11].

SGLT2 is currently the focus of interest as a potential therapeutic target for reducing hyperglycemia in T2DM, and several selective SGLT2 inhibitors have been developed [12-16]. In diabetic animal models, pharmacological inhibition of SGLT2 leads to glucosuria, and improvement of plasma glucose levels, followed by a reduction of insulin resistance [17-19].

SGLT2 inhibitors have the potential to offer distinct advantages over currently available diabetic treatments. Because SGLT2 inhibitors work by an insulin-independent mechanism, this class of compounds may be of benefit as adjunctive therapy in patients whose pancreatic function is diminished or in patients who have insulin resistance. Thus, treatment with SGLT2 inhibitors may be appropriate in all stages of T2DM, provided the patient still has adequate renal function to deliver the drug to the site of action in the kidney. Another advantage is that SGLT2 inhibitors cause calorie wasting by loss of glucose in the urine, thus offering the potential for promoting weight loss, whereas some other anti-diabetic treatments such as sulfonylureas and insulin promote weight gain.

**Figure 1:**
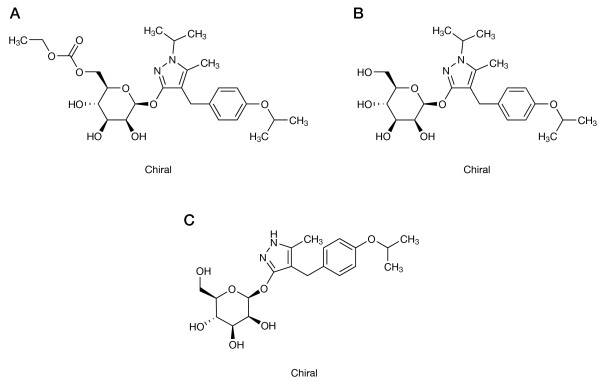
**Structures of remogliflozin etabonate, remogliflozin, and GSK279782.** Structures of (**A**) remogliflozin etabonate, (**B**) remogliflozin and (**C**) GSK279782).

Remogliflozin etabonate is the ester prodrug of remogliflozin [20], which is the active entity that selectively inhibits SGLT2. Remogliflozin undergoes further transformation to GSK279782, an active metabolite. The structures of remogliflozin etabonate, remogliflozin and GSK279782 are presented in Figure 1.

Remogliflozin etabonate causes a concentration-dependent increase in urinary glucose excretion in mice and rats [20,21]. Unlike earlier SGLT inhibitors, such as phlorizin and T-1095, remogliflozin displays a high level of selectivity for SGLT2 over SGLT1 [22]. This single-dose evaluation was the first study to be conducted with remogliflozin etabonate in humans and was designed to provide safety, tolerability, PK and pharmacodynamic information.

## Methods

This single center study was conducted at Profil Institute for Clinical Research (Chula Vista, CA, USA) and was conducted in accordance with Good Clinical Practice and the principles of the Declaration of Helsinki. The study protocol and subject information were reviewed and approved by Biomedical Research Institute of America investigational reviewer board (San Diego, CA, USA) and all subjects provided written, informed consent prior to start of study-related procedures.

### Subjects

Ten healthy male and female subjects followed by a separate group of 6 subjects with T2DM were enrolled in this study. Enrollment of women was restricted to those who were postmenopausal or surgically sterile. All subjects gave written informed consent prior to participation in any study-related procedures. Healthy subjects were required to be 18–55 years of age, and have a body mass index (BMI) of 19.0 to 30.0 kg/m^2^ inclusive. Subjects with T2DM were required to be 30–60 years of age, have a BMI of 22–35 kg/m^2^, to be healthy other than having been diagnosed with T2DM at least 6 months prior to entry in the study, and to have been maintained on a stable treatment regimen for at least 3 months. Diabetic subjects were required to have hemoglobin A1c (HbA1c) ≤10% and fasting plasma glucose <280 mg/dL at screening. Participants with diabetes were required to be on a stable treatment regimen using a single oral antidiabetic agent (either sulfonylureas, rosiglitazone, metformin or acarbose) or management by diet and exercise. All T2DM subjects also had to be willing and medically able to discontinue their diabetes medications for up to 72 h during each treatment period. Subjects were excluded if they had been taking diuretics, corticosteroids or other medications that might result in electrolyte depletion; had required insulin during the last 3 months; had significant renal disease; or if their participation would have resulted in donation of blood in excess of 550 mL within an 8-week period.

### Study design

This double-blind, randomized, placebo-controlled, single escalating-dose crossover study was conducted in two parts: Part A consisted of a randomized, dose-escalation in healthy subjects; Part B was of similar design, but conducted in subjects with T2DM and included an evaluation of pharmacodynamics using a 50 g oral glucose tolerance test (OGTT).

#### Part A

Ten healthy subjects were evaluated in 5 study sessions, each separated by approximately 2 weeks. At each study session, subjects received either an oral dose of remogliflozin etabonate or placebo after an overnight fast. Remogliflozin etabonate doses were 20 mg, 50 mg, 150 mg, 500 mg and 1000 mg. Over the course of participation in the study, each subject received 4 of the 5 active remogliflozin etabonate doses and 1 dose of placebo (4:1 active to placebo ratio per treatment period). The available safety and PK results from each dosing period were evaluated before proceeding to the next dose level.

#### Part B

Six subjects with T2DM received two doses of remogliflozin etabonate and a placebo dose, in a randomized, dose escalating, crossover design, along with an oral glucose load on three study sessions separated by 7–14 days. Full PK and safety profiles were measured on Day 1 of each dosing period. The doses selected for this portion of the study, 50 mg and 500 mg, were based on data obtained in Part A. In each dosing period, subjects were assigned to active vs placebo in a 2:1 ratio.

All subjects were admitted to the unit two nights prior to receiving study drug to establish baseline safety parameters and fluid intake levels over a 36 h period. Subjects remained in the unit for at least 24 h after doses were administered for monitoring of clinical laboratory parameters exploratory biomarkers, vital signs, ECGs and adverse events. While confined to the clinical research unit, all subjects received meals standardized with respect to calories, fat, protein, carbohydrate, and sodium content; however, detailed dietary information was not captured in this study. In Part A, subjects were dosed following an overnight fast; lunch and dinner were provided at 4 and 10 h after dosing, respectively. Fifteen minutes after dosing in each treatment period in Part B, a fasting OGTT was performed using 50 g glucose (administered as 50 g Glucola™). A 50 g glucose load was chosen since the OGTT was being performed in subjects already known to have diabetes. The glucose drink was consumed by subjects within approximately 5 minutes. Blood samples for the measurement of glucose, insulin, and intact glucagon-like peptide 1 (GLP-1) were collected for 24 hours following dose administration. Provided that there were no safety or tolerability concerns, subjects were released from the clinic on day 2 of each treatment period until their return for the next treatment or follow-up period. Each subject was involved in the study for approximately 8 weeks (from screening to follow-up).

### Pharmacokinetic assessments

#### Blood collections and analysis

On each dosing day, a series of 2.0 mL blood samples were collected at pre-dose and 10, 20, 30 and 45 min, and 1, 1.25, 1.5, 2, 2.5, 3, 4, 6, 8, 12, 16, and 24 h post-dose for the determination of remogliflozin etabonate, remogliflozin and GSK279782 in plasma by using high-performance liquid chromatography with tandem mass spectrometry (MS/MS) as described [23].

#### Pharmacokinetic calculations

Non-compartmental PK analysis of plasma concentration–time data was performed using WinNonlin Version 4.1 (Pharsight Corporation, Mountainview, CA, USA). The C_max_ and T_max_ were obtained directly from the data. Areas under the plasma concentration–time curves from time zero to the last quantifiable time point (AUC_[0–last]_) and extrapolated to infinity (AUC_[0–∞]_) were calculated using the log-linear trapezoidal method. The terminal plasma elimination rate-constant (λz) was estimated from log-linear regression analysis of the terminal phase of the plasma concentration–time curve, and the T_½_ was calculated as T_½_ = ln2/λz. Ratios of AUC_(0–∞)_ remogliflozin to AUC_(0–last)_ remogliflozin etabonate were calculated including molecular weight corrections.

### Pharmacodynamic assessments

#### Plasma pharmacodynamics

Blood samples for glucose were taken at 0 (pre-dose), and 1, 2, 4, 8 and 12 h after dosing in each treatment period (Part A). For Part B, blood samples for glucose, insulin and GLP-1 were taken at check-in on day -2, and at 0 h (pre-dose), and 0.5, 1, 1.5, 2, 4 h (prior to lunch), 4.5, 5, 6, 8, 10, 12 and 24 h after dosing on day 1 of each treatment period.

##### Glucose and insulin sample handling

For glucose, plasma was analyzed using a YSI 2300 Glucose Analyzer (Yellow Springs International Life Sciences, Yellow Springs, OH, USA). For insulin, plasma was rapidly prepared and frozen at -70°C until analyzed by LabCorp (San Diego, CA, USA) using a chemiluminescent immunometric assay method (Siemen’s Immulite 2000 analyzer with Immulite Insulin Kit L2KIN2).

##### GLP-1 sample handling

For assay of intact GLP-1, blood was collected into a chilled EDTA tube and protease inhibitors (DPP4 inhibitor obtained from EMD Millipore, St. Charles, MO) were immediately added. Samples were then spun down and plasma split into two separate tubes and frozen at -70°C until analyzed by Pathway Diagnostics (Malibu, CA, USA) by ELISA (kit # EGLP-35 K, EMD Millipore). The lowest level of intact GLP-1 this assay can detect is 2 pM (with a minimum plasma sample size of 0.4 mL).

### Urine pharmacodynamics

#### Sample collection

Urine samples were collected at pre-dose, and over a series of intervals (0–2, 2–4, 4–6, 6–8, 8–12 and 12–24 h post-dose) for the analysis of creatinine, glucose and electrolytes (Na, K and Cl). All fluid intake was recorded, as well as urine volume, over the 24 hours before and after dosing in each treatment period. Urine was tested for protein on the first morning void of day -1 and 24 h after dosing.

#### Calculations

Creatinine clearance (CL_CR_) was calculated as the amount of creatinine excreted in 24 h (Ae_0–24 h_) divided by the mean of the pre-dose and 24-h post-dose plasma creatinine levels. The percentage of filtered glucose excreted in the urine for each individual time period was calculated as the amount of glucose excreted during that time period divided by (CL_CR_ × PG × time interval length), where CL_CR_ is the creatinine clearance for the time interval, PG is the plasma glucose concentration closest to the midpoint of the time interval, and the time interval is the period (min) of urine collection (CL_CR_ × PG represents the glucose filtered load). For the 24-h period, the percentage of filtered glucose excreted was calculated as the amount of glucose excreted over 24 h divided by the sum over the individual time intervals of (CL_CR_ × PG × time interval length).

### Statistical analysis

Safety and pharmacodynamic data were summarized using descriptive statistics. This was a small exploratory study and no formal hypothesis-testing was conducted. Dose proportionality with respect to C_max_, and AUC was assessed using the power model y = α dose^β^, where y = C_max_ or AUC, and α denotes a random subject effect. The exponent β in the power model will be estimated by regressing the log_e_-transformed PK parameters on log_e_ dose, i.e. ln(PK parameter) = ln(α) + β * ln(dose). Dose proportionality implied that β = 1 and was assessed by estimating β and its corresponding 90% confidence interval. The power model was fitted by restricted maximum likelihood using SAS Proc Mixed.

## Results

### Subject demographics

In Part A, 10 subjects (8 males, 2 females, mean age of 39 years, mean BMI of 24.5 mg/kg^2^, and mean baseline fasting plasma glucose 4.7 mmol/L [range 4.2 to 5.1 mmol/L, SD 0.29 mmol/L]) were randomized; 9 completed the study (1 subject participated in all the study visits but did not return for the follow up visit). In Part B, 6 T2DM subjects (2 males, 4 females, mean age of 53 years, mean BMI of 30.5 mg/kg^2^, and mean baseline fasting plasma glucose 8.9 mmol/L [range 5.81 to 12.1 mmol/L, SD 2.47 mmol/L]) were randomized and all completed the dosing period.

### Safety and tolerability

Remogliflozin etabonate was generally well-tolerated by all subjects. There were no obvious patterns suggesting an effect of remogliflozin etabonate on clinical laboratory results, urine electrolytes, vital signs or ECGs. There were no deaths, serious adverse events or adverse events (AEs) leading to withdrawal. The most frequently reported AE was headache. AEs are summarized in Table 1.

**Table 1:** Summary of adverse events

		**Remogliflozin etabonate dose**					
	**Placebo**	**20 mg**	**50 mg**	**150 mg**	**500 mg**	**1000 mg**	**Total**
**Healthy Subjects**	**n = 10**	**n = 8**	**n = 8**	**n = 8**	**n = 8**	**n = 8**	**n = 10**
	**n (%)**	**n (%)**	**n (%)**	**n (%)**	**n (%)**	**n (%)**	**n (%)**
At least one adverse event	4 (40)	2 (25)	3 (38)	1 (13)	3 (38)	1 (13)	8 (80)
**Adverse events reported by >1 subject in total**							
Headache	1 (10)	1 (13)	1 (13)	0	2 (25)	1 (13)	4 (40)
Blood creatine phosphokinase increased	1 (10)	0	0	0	1 (13)	0	2 (20)
**Drug-related adverse events**							
Headache	1 (10)	1 (13)	1 (13)	0	0	1 (13)	3 (30)
Dizziness	0	0	0	0	0	1 (13)	1 (10)
Diarrhea	0	1 (13)	0	0	0	0	1 (10)
Hot flush	0	0	0	0	0	1 (13)	1 (10)
**T2DM subjects**	**n = 6**		**n = 6**		**n = 6**		**n = 6**
	**n (%)**		**n (%)**		**n (%)**		**n (%)**
At least one adverse event	3 (50)		1 (17)		2 (33)		4 (67)
**Adverse events reported by >1 subject in total**							
Muscle cramp	1 (17)		1 (17)		0		2 (33)
β_2_-microglobulin increased	0		0		2 (33)		2 (33)
**Drug-related adverse events**							
Nausea	0		0		1 (17)		1 (17)
β_2_-microglobulin increased	0		0		1 (17)		1 (17)
Pain in extremity	0		0		1 (17)		1 (17)

n = number of subjects reporting event.

% = percentage of subjects reporting event.

Urine beta-2 microglobulin levels, an exploratory biomarker that was measured as a potential early indicator of renal toxicity, were within the normal range at both baseline and after treatment for all subjects except for two subjects with diabetes.

One of these subjects had normal beta-2 microglobulin values at 1 and 4 days after dosing with 500 mg remogliflozin etabonate. However, 11 days after dosing, this subject returned to the clinic for the Day -2 visit of the 3^rd^ treatment period. At this time, the subject’s beta-2 microglobulin levels were elevated to 2.5 μg/mL. The values returned to normal (<0.3 μg/mL) within 2 days. The investigator attributed the elevated pre-placebo levels to other concomitant disease. A second subject, however, did have what was considered by the investigator to be a drug-related elevation of beta-2 microglobulin of 1.62 μg/mL on day 1 after dosing with 500 mg remogliflozin etabonate. The value returned to normal levels within 4 days. No associated changes in serum creatinine and urea or urine microalbumin were observed.

### Pharmacokinetics

#### Healthy subjects

**Figure 2:**
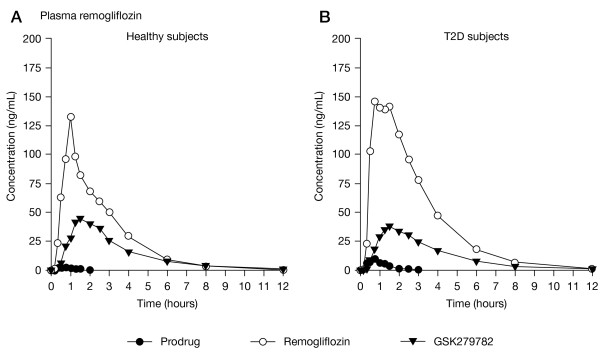
Mean plasma concentration-time profiles for remogliflozin etabonate (prodrug), remogliflozin (active entity), and GSK279782 (metabolite) following a 50 mg dose of remogliflozin etabonate to subjects.

PK parameters are summarized in Table 2 (remogliflozin etabonate), and Table 3 (remogliflozin and GSK279782). Remogliflozin etabonate was rapidly absorbed and extensively hydrolyzed to the active entity in all dose groups. The median T_max_ estimates for the prodrug ranged from 0.52 to 1.25 h and median T_½_ estimates ranged from 0.26 to 0.71 h. The concentration–time profiles are shown for the 50 mg dose in Figure 2 illustrating low circulating plasma concentrations of prodrug relative to active entity.

**Table 2:** **Summary of plasma remoglifozin etabonate pharmacokinetic parameters in healthy subjects**^**a,b**^

**Remogliflozin etabonate dose**	**20 mg**	**50 mg**	**150 mg**	**500 mg**	**1000 mg**
**AUC**_**(0–∞) **_**(ng•h/mL)**	NQ^c^	3.70 (46)^d^	9.77 (48)^d^	36.8 (67)^e^	126 (44)^f^
**AUC**_**(0–t) **_**(ng.h/mL)**	1.61 (67) ^g^	3.56 (56)	9.51 (42)	35.4 (62)	107 (53)
**C**_**max **_**(ng/mL)**	1.89 (78)	4.98 (61)	17.6 (48)	41.6 (81)	144 (59)
**T**_**max **_**(h)**	0.625	0.625	0.515	1.25	0.625
	0.33-2.03	0.17-1.50	0.17-1.50	0.33-2.50	0.33-2.50
**T**_**½ **_**(h)**	NQ^c^	0.353 (56)^d^	0.256 (35)^d^	0.263^e^ (27)	0.707^f^ (56)

a. Geometric mean and CV% except for T_max_ (median and range).

b. n = 8 unless otherwise specified.

c. NQ, not quantifiable because concentrations at later time points were below the limit of quantification.

d. n = 6.

e. n = 4.

f. n = 7.

g. n = 5.

**Table 3:** **Summary of plasma remogliflozin and GSK279782 PK parameters in healthy subjects**^**a**^

**Remogliflozin etabonate dose**	**20 mg**		**50 mg**		**150 mg**		**500 mg**		**1000 mg**	
**Analyte**	**R**^**b**^	**279782**	**R**^**b**^	**279782**	**R**^**b**^	**279782**	**R**^**b**^	**279782**	**R**^**b**^	**279782**
**AUC**^c^_**(0-∞) **_**(ng•h/mL)**										
Geometric mean (CV%)	133 (45)	51.8 (48)	324 (29)	145 (48)	991 (25)	447 (44)	3721 (29)	1523 (40)	10257 (17)	3995 (22)
**C**_**max **_**(ng/mL)**										
Geometric mean (CV%)	61 (54)	17.5 (72)	158 (44)	50.2 (62)	515 (37)	155 (50)	1703 (45)	498 (44)	4822 (37)	1286 (28)
**T**_**max **_**(h)**										
Median	0.89	1.26	1.14	1.38	0.66	1.00	1.50	1.50	1.25	1.25
(range)	(0.50–1.5)	(0.75–2.5)	(0.50–1.5)	(1.00–2.0)	(0.33–2.0)	(0.75–2.0)	(0.50–3.0)	(1.00–4.0)	(0.50–3.0)	(0.75–3.0)
**T**_**½ **_**(h)**										
Geometric mean (CV%)	1.38 (21)	1.54 (11)	1.47 (15)	2.19 (17)	1.59 (13)	2.28(13)	2.57 (29)	3.07 (13)	2.86 (17)	3.50 (12)
**AUC ratio (remogliflozin/remogliflozin etabonate)**										
Geometric mean	84^d^	—	81	—	102	—	105	—	95	—
CV%	(39)		(34)		(53)		(86)		(69)	

a. n = 8 unless otherwise specified.

b. R = remogliflozin.

c. The median percentage of AUC(0-∞) extrapolated for R was low ranging from 0.07% to 2.1% across all doses and for 279782 ranging from 0.29% to 4.51%.

d. n = 5.

Remogliflozin appeared in plasma at relatively high concentrations within 10 min of remogliflozin etabonate dosing. Mean remogliflozin C_max_ of the active entity occurred within 1.5 h of prodrug dosing and was eliminated from plasma more slowly than prodrug. The mean T_½_ estimates ranged from 1.38 to 2.86 h. The AUC(0-∞) ratios of remogliflozin to prodrug (or AUC(0-t) when AUC(0-∞) not available for prodrug), ranged from 81 to 105 indicating extensive conversion to active entity. The ratios were consistent across all doses.

An active metabolite of remogliflozin, GSK279782, was monitored in this study and found to be present in relatively high concentrations in plasma. An estimated mean AUC(0-∞) ratio of GSK279782 to remogliflozin was 40 to 45%. The median T_max_ estimates for GSK279782 ranged from 1.0 to 1.5 h and median T_½_ estimates ranged from 1.54 to 3.50 h. The concentration profiles of GSK279782 followed a similar time course to those of remogliflozin with only a small delay in appearance of C_max_ and slightly longer T_½_.

The statistical analysis showed AUC(0-∞) and C_max_ for all three analytes increased nearly dose proportionally over the 50-fold dose range of 20 to 1000 mg in healthy subjects. For remogliflozin etabonate, the mean slopes and 90% confidence intervals (CI) for AUC(0-∞) and Cmax were 1.17 (1.04, 1.30) and 1.04 (0.94, 1.14). For remogliflozin, the mean slopes and 90% CIs for AUC(0-∞) and Cmax were 1.09 (1.06, 1.12) and 1.08 (1.02, 1.14). For GSK279782, the mean slopes and 90% CIs for AUC(0-∞) and Cmax were 1.08 (1.05, 1.12 and 1.07 (1.01, 1.12).

#### Type 2 diabetes subjects

The PK parameters for remogliflozin etabonate, remogliflozin, and GSK279782 in subjects with T2DM are shown in Table 4. The prodrug was rapidly absorbed and extensively hydrolyzed to the active entity in patients as well as non-diabetic subjects. There were no discernable differences in mean T_max_ or T_½_ values of remogliflozin or GSK279782 between patients and healthy subjects. The apparent differences in AUCs for remogliflozin and AUC ratios between populations are potentially the result of small sample size and variability rather than differences in absorption or metabolism.

**Table 4:** **Summary of plasma remogliflozin etabonate, remogliflozin, and GSK279782 PK parameters in T2DM subjects**^**a**^

	**Remogliflozin etabonate**		**Remogliflozin**		**GSK279782**	
**Remogliflozin etabonate dose**	**50 mg**	**500 mg**	**50 mg**	**500 mg**	**50 mg**	**500 mg**
**AUC**_**(0–∞)**_^**c**^**(ng•h/mL)**						
Geometric mean (CV%)	8.91 (58)	91.9 (46) ^b^	523 (38)	5176 (44)	130 (50)	1293 (45)
**C**_**max **_**(ng/mL)**						
Geometric mean (CV%)	9.56 (39)	83.9 (89)	195 (46)	1891 (49)	34.6 (39)	314 (39)
**T**_**max **_**(h)**						
Median	0.58	0.75	1.46	2.50	1.74	2.75
(range)	(0.33–0.78)	(0.33–2.50)	(0.33–2.00)	(0.33–4.00)	(0.75–4.00)	(0.75–4.00)
**T**_**½ **_**(h)**						
Geometric mean (CV%)	0.61 (32)	0.82 (69) ^b^	1.59 (27)	3.93 (25)	2.05 (24)	3.28 (23)
**AUC**_**(0–∞) **_**ratio**						
**(Remogliflozin/remogliflozin etabonate)** Geometric mean (CV%)	—	—	59 (62)	54 (38)	—	—

a. n = 6 unless otherwise specified.

b. n = 2.

c. The median percentage of AUC(0-∞) extrapolated for remogliflozin etabonate ranged from 10% to 11% and for remogliflozin was low ranging from 0.14% to 0.42% and for GSK279782 was 0.47% to 2.0%.

### Pharmacodynamics

#### Urine glucose

**Figure 3:**
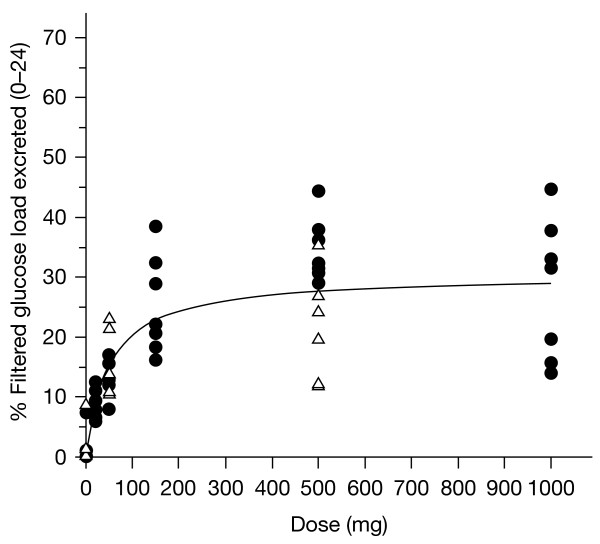
The filtered glucose load (%) vs dose in healthy volunteers (filled circles) and subjects with T2DM (open triangles).

**Figure 4:**
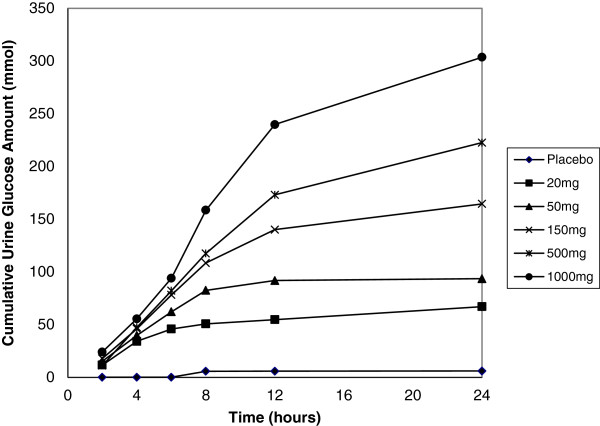
Mean cumulative 24-h urine glucose excretion following single-dose administration of remogliflozin etabonate (20 to 1000 mg) in healthy subjects.

In both populations, the total amount of glucose excreted in urine from 0–24 h increased in a dose-dependent manner; however, urine glucose excretion increased less than proportionally with increasing doses of remogliflozin etabonate (Table 5), suggesting a plateau of effect. The urine glucose excretion was higher in the T2DM subjects due to higher plasma glucose concentrations. When urine glucose excretion was corrected for circulating plasma glucose concentrations and CL_CR_ (to provide an estimate of percentage filtered glucose load or FGL%), the FGL% was similar between the populations. Figure 3 illustrates the similarity between populations and the saturation of urine glucose excretion with increasing doses. The saturation is related to maximal SGLT2 transporter inhibition. Figure 4 shows the cumulative mean values for the amount of glucose excreted in healthy subjects over time for each dose.

**Table 5:** 24-h urinary glucose and electrolyte excretion, after single-dose administration of remogliflozin etabonate (20–1000 mg) in healthy volunteers (n = 8 per remogliflozin etabonate group and n = 10 for placebo) and subjects with T2DM (n = 6)

**Parameter**	**Placebo**	**Remogliflozin etabonate dose, mg**				
		**20**	**50**	**150**	**500**	**1000**
**Healthy subjects**						
Urinary glucose excretion (mmol)	6.5 (18.6)^a^	67.1 (17.9)	96.7 (17.1)^b^	168 (49.4)^b^	223 (49.5)	304 (137)
Filtered glucose excreted in urine (%)	0.9 (2.4)^a^	9.0 (2.2)	12.7 (3.7)^b^	25.5 (7.8)^b^	34.2 (5.0)	26.4 (11.7)
Urinary sodium excretion (mmol)	162 (49.4)^a^	148 (64.0)	212 (67.6)^b^	176 (38.7)^b^	143 (55.0)	207 (62.9)
Urinary chloride excretion (mmol)	141 (39.8)^a^	136 (56.6)	189 (51.4)^b^	179 (49.6)^b^	126 (55)	201 (61.5)
Urinary potassium excretion (mmol)	62.2 (15.7)^a^	66.6 (27.7)	75.2 (21.3)^b^	59.7 (20.3)^b^	55.8 (17.2)	89.1 (24.7)
**T2DM subjects**						
Urinary glucose excretion (mmol)	40.4 (62.4)^c^		384 (210)		642 (256)	
Filtered glucose excreted in urine (%)	2.3 (3.6)^c^		15.9 (5.9)^c^		21.6 (9.1)	
Urinary sodium excretion (mmol)	196 (39.2)^c^		173 (35.5)^c^		301 (128)	
Urinary chloride excretion (mmol)	181 (48.3)^c^		168 (23.9)^c^		287 (147)	
Urinary potassium excretion (mmol)	71.7 (12.1)^c^		65.8 (4.5)^c^		108 (65.8)	

Results are expressed as mean (SD).

a. n = 9.

b. n = 7.

c. n = 5.

#### Urine electrolytes

Urine electrolytes are also summarized in Table 5. Urine excretion of electrolytes was highly variable, and no treatment-related changes were observed. Much of the variability in the 500 mg dose period can be attributed to one subject (#12) whose values for all three electrolytes were roughly 2-fold higher than those of the other participants during that period.

#### Plasma glucose and insulin

For healthy subjects, AUC values for plasma glucose were very similar between placebo and remogliflozin etabonate periods at both 0–4 h (mean of approximately 18 mmol•h/L) and 0–12 h (mean of approximately 55–59 mmol•h/L) following study drug administration.

**Figure 5:**
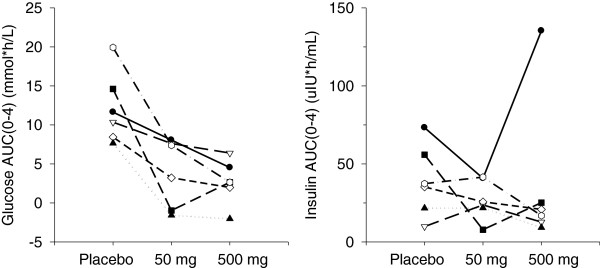
**Plasma glucose and insulin AUC**_**0-4 h **_**following glucose challenge in subjects with T2DM.**

For T2DM subjects, the increases in plasma glucose following an OGTT were clearly attenuated between placebo and 50 mg RE; the effect on glucose was altered little by increasing the dose from 50 mg to 500 mg RE. There were no clear trends in plasma insulin following the glucose load. The baseline adjusted AUC_(0–4)_ values for plasma glucose and insulin following an OGTT, are depicted in Figure 5.

#### Plasma intact GLP-1

In T2DM subjects, the median baseline adjusted AUC_(0–4)_ for plasma GLP-1 following an OGTT was 6.36 pM*h (range, 0 to 16.6) for placebo, -8.85 pM*h (range, -46.7 to 33.4) for remogliflozin etabonate 50 mg, and -1.95 pM*h (range, -11.6 to 7.0) for remogliflozin etabonate 500 mg. These data are difficult to interpret because the ranges for placebo, 50 mg, and 500 mg groups contain zero, and also because the analytical method likely did not include extraction of the samples, which can confound the analysis of intact GLP-1 [24,25].

### Fluid balance

Fluid balance data for both healthy and T2DM subjects are shown in Table 6.

**Table 6:** Summary of fluid balance data (mL)

		**Remogliflozin etabonate dose**				
	**Placebo**	**20 mg**	**50 mg**	**150 mg**	**500 mg**	**1000 mg**
**Healthy Subjects**	**(n = 10)**	**(n = 8)**	**(n = 8)**	**(n = 8)**	**(n = 8)**	**(n = 8)**
-24 h to pre-dose	-462 (605)	-460 (1311)	-338 (525)	-512 (731)	-278 (1250)	-126 (798)
0–12 h post-dose	163 (668)	243 (1161)	-570 (582)	-86 (909)	-159 (531)	-273 (331)
12–24 h post-dose	-739 (423)	-581 (387)	-551 (530)	-564 (478)	-424 (334)	-495(367)
**T2DM subjects**	**n = 6**		**n = 6**		**n = 6**	
-24 h to pre-dose	-312 (457)		-504 (294)		188 (1879)	
0–12 h post-dose	569 (400)		-78(433)		-29 (749)	
12–24 h post-dose	-908 (524)		-803 (658)		-1088(679)	

Fluid balance = total fluid intake minus total urine volume.

Results are expressed as mean (SD).

On the day prior to dosing in healthy subjects, fluid balance (total fluid intake minus total urine volume) was negative (mean volumes in the range of -126 to -512 mL). In the 0–12 h interval after dosing, fluid balance shifted to positive for the placebo (+163 mL) and 20 mg remogliflozin etabonate (+242 mL) periods, while the 50 to 1000 mg remogliflozin etabonate treatment periods remained negative. For the 12 to 24 h post-dose interval, all regimens had a fluid balance that was negative. There was no evidence of a clear dose–response.

In T2DM subjects, fluid balance was variable in the 24 h prior to dosing; T2DM subjects assigned to placebo and remogliflozin etabonate 50 mg had a negative fluid balance (mean volumes ranging from -312 mL to -504 mL), while those assigned to remogliflozin etabonate 500 mg had a positive fluid balance (mean volume +188 mL). From 0–12 h after dosing, fluid balance was positive for the placebo regimen and negative for the remogliflozin etabonate 50 mg and 500 mg regimens. For the 12–24 h interval, all regimens had negative fluid balance (mean volumes ranging from -802 mL to -1087 mL). As with the healthy subjects, there was no clear dose–response.

## Discussion

By promoting urinary glucose excretion, SGLT2 inhibitors offer a novel mechanism of antidiabetic action that is complementary to currently available classes of drugs which reduce hepatic gluconeogenesis (e.g. metformin), increase glucose flux into muscle and fat (e.g. insulin sensitizers such as thiazolidinediones) or stimulate β-cell insulin secretion (e.g. GLP-1-based therapies).

In both healthy and T2DM subject populations, the prodrug, remogliflozin etabonate was extensively converted to its active entity, remogliflozin, and to an active metabolite, GSK279782, which is as potent as remogliflozin in inhibiting SGLT-2 in vitro [23]. The PK parameters (C_max_ and AUC) of all three analytes (remogliflozin etabonate, remogliflozin, and GSK279782) increased nearly dose proportionally over the 50-fold dose range, 20 to 1000 mg, of remogliflozin etabonate in healthy subjects. Although limited data are available, there appeared to be dose proportionality in subjects with T2DM between 50 and 500 mg doses as well. The PK parameters were similar between the healthy subjects and T2DM subjects.

Evidence of the desired pharmacological effect was seen in the dose-dependent increase in urinary glucose excretion in healthy subjects and in T2DM subjects following administration of remogliflozin etabonate. The total amount of glucose excreted in the 24 h after dosing increased less than proportional to the dose and exposure to remogliflozin in healthy subjects. The plateau in glucose excretion was observed between exposures associated with single doses of 150 mg and 500 mg, suggesting maximal inhibition of the SGLT2 transporter at this dose range. Subjects with T2DM showed increased glucose excretion over 24 hours with increasing doses of remogliflozin etabonate, but the percentage of filtered glucose excreted was similar between the two study groups. This amount of urine glucose excretion has been predicted to provide clinically meaningful reduction in plasma glucose [26]. For subjects with T2DM, the increase in plasma glucose following an oral glucose load appeared to be blunted in a drug-dependent fashion following dosing with remogliflozin etabonate, suggesting that SGLT2 inhibition is an appropriate mechanism for glucose lowering in these patients.

## Conclusions

This single-dose evaluation is the first clinical study to be conducted with remogliflozin etabonate, and it provides safety, tolerability, pharmacokinetic and pharmacodynamic information in both healthy subjects and those with T2DM. In this study, single oral doses of remogliflozin etabonate (20 mg to 1000 mg for healthy subjects; 50 mg and 500 mg for subjects with T2DM) were generally safe and well-tolerated. Clinically significant fluid and electrolyte imbalances were not seen in this study, but longer repeat dose studies will be required to establish the safety profile. Remogliflozin etabonate increased urinary glucose excretion in a dose dependent fashion and also blunted increases in plasma glucose following an oral glucose load, which suggests that remogliflozin etabonate could be useful as a treatment for T2DM.

## Competing interests

AK, ROCS, EKH, RLD, WT, GAS, JWP, CJ, and DN are/were all employees of GlaxoSmithKline at the time of the study; IM is an employee of Kissei Pharmaceutical Company.

## Authors’ contributions

AK, ROCS, EKH, RLD, WT, GAS, JWP, CJ, and DN participated in the design of the study, its co-ordination and performed the statistical analysis. IM contributed to study design and the analysis, and MH contributed to study conduct. All authors have been involved in critically revising the drafts of the manuscript and read and approved the final manuscript.

## Pre-publication history

The pre-publication history for this paper can be accessed here:

http://www.biomedcentral.com/2050-6511/14/26/prepub
